# Contrast-enhanced CT radiomics combined with multiple machine learning algorithms for preoperative identification of lymph node metastasis in pancreatic ductal adenocarcinoma

**DOI:** 10.3389/fonc.2024.1342317

**Published:** 2024-09-13

**Authors:** Yue Huang, Han Zhang, Lingfeng Chen, Qingzhu Ding, Dehua Chen, Guozhong Liu, Xiang Zhang, Qiang Huang, Denghan Zhang, Shangeng Weng

**Affiliations:** ^1^ Department of Hepatopancreatobiliary Surgery, The First Affiliated Hospital of Fujian Medical University, Fuzhou, Fujian, China; ^2^ Fujian Abdominal Surgery Research Institute, The First Affiliated Hospital of Fujian Medical University, Fuzhou, Fujian, China; ^3^ National Regional Medical Center, Binhai Campus of the First Affiliated Hospital, Fujian Medical University, Fuzhou, Fujian, China; ^4^ Department of Radiology, The First Affiliated Hospital of Fujian Medical University, Fuzhou, Fujian, China; ^5^ Fujian Provincial Key Laboratory of Precision Medicine for Cancer, The First Affiliated Hospital of Fujian Medical University, Fuzhou, Fujian, China; ^6^ Clinical Research Center for Hepatobiliary Pancreatic and Gastrointestinal Malignant Tumors Precise Treatment of Fujian Province, The First Affiliated Hospital of Fujian Medical University, Fuzhou, Fujian, China

**Keywords:** pancreatic ductal adenocarcinoma, radiomics, lymph node metastasis, machine learning, computed tomography

## Abstract

**Objectives:**

This research aimed to assess the value of radiomics combined with multiple machine learning algorithms in the diagnosis of pancreatic ductal adenocarcinoma (PDAC) lymph node (LN) metastasis, which is expected to provide clinical treatment strategies.

**Methods:**

A total of 128 patients with pathologically confirmed PDAC and who underwent surgical resection were randomized into training (n=93) and validation (n=35) groups. This study incorporated a total of 13 distinct machine learning algorithms and explored 85 unique combinations of these algorithms. The area under the curve (AUC) of each model was computed. The model with the highest mean AUC was selected as the best model which was selected to determine the radiomics score (Radscore). The clinical factors were examined by the univariate and multivariate analysis, which allowed for the identification of factors suitable for clinical modeling. The multivariate logistic regression was used to create a combined model using Radscore and clinical variables. The diagnostic performance was assessed by receiver operating characteristic curves, calibration curves, and decision curve analysis (DCA).

**Results:**

Among the 233 models constructed using arterial phase (AP), venous phase (VP), and AP+VP radiomics features, the model built by applying AP+VP radiomics features and a combination of Lasso+Logistic algorithm had the highest mean AUC. A clinical model was eventually constructed using CA199 and tumor size. The combined model consisted of AP+VP-Radscore and two clinical factors that showed the best diagnostic efficiency in the training (AUC = 0.920) and validation (AUC = 0.866) cohorts. Regarding preoperative diagnosis of LN metastasis, the calibration curve and DCA demonstrated that the combined model had a good consistency and greatest net benefit.

**Conclusions:**

Combining radiomics and machine learning algorithms demonstrated the potential for identifying the LN metastasis of PDAC. As a non-invasive and efficient preoperative prediction tool, it can be beneficial for decision-making in clinical practice.

## 1 Introduction

Pancreatic ductal adenocarcinoma (PDAC) accounts for the seventh-highest cancer mortality rate worldwide, and 50% of patients with PDAC suffer from metastatic disease (1). Recently, neoadjuvant therapy has emerged as a significant component in the management of PDAC (2). According to an investigation, the administration of neoadjuvant therapy followed by resection is associated with improved rates of overall survival in comparison to individuals who undergo upfront resection followed by adjuvant therapy (3). Neoadjuvant chemotherapy for pancreatic cancer with significant lymph node (LN) metastases was recommended by the National Comprehensive Cancer Network (NCCN) guidelines (4). Hence, the preoperative evaluation of LN status holds significant importance in establishing a personalized treatment strategy for individuals diagnosed with PDAC.

The most commonly used imaging modality to evaluate patients with PDAC prior to surgery is computed tomography (CT). However, previous studies have suggested that the ability of CT to effectively forecast the presence of nodal involvement in surgically treatable PDAC is constrained (5). Approximately 70% of patients with negative clinical lymph nodes present with lymph node involvement upon pathologic assessment (6). The lymph node enlargement may occur independently of localized inflammation caused by malignant biliary obstruction (7). This indicates that the preoperative radiological staging of nodule involvement remains difficult. Therefore, there is an unquestionable need for more effective methods to assess the LN status for patients with pancreatic cancer.

Radiomics provides a large amount of medical image information that can reveal hidden features of diseases that are invisible to the naked eye (8). Previous research has demonstrated the efficacy of integrating radiomics and machine learning (ML) techniques in many applications (9–11). Several researchers have constructed multiphasic contrast-enhanced CT (CECT) radiomics models to evaluate the preoperative LN status of PDAC (12–14). These studies indicated that radiomics models have significant potential in predicting pancreatic cancer with lymph node metastasis (LNM). However, the researchers predominantly chose modeling algorithms based on their preferences and limitations in knowledge. It is imperative that evidence be provided to select appropriate models for solving clinical problems. To the best of our knowledge, no study has explored the use of radiomics combined with multiple ML algorithms in the preoperative identification of the LN status in PDAC. The objective of this study was to evaluate the predictive value of clinical data and radiomic features for forecasting the LN status of patients with pancreatic cancer using a combination of various ML algorithms.

## 2 Materials and methods

### 2.1 Patients

**Figure 1:**
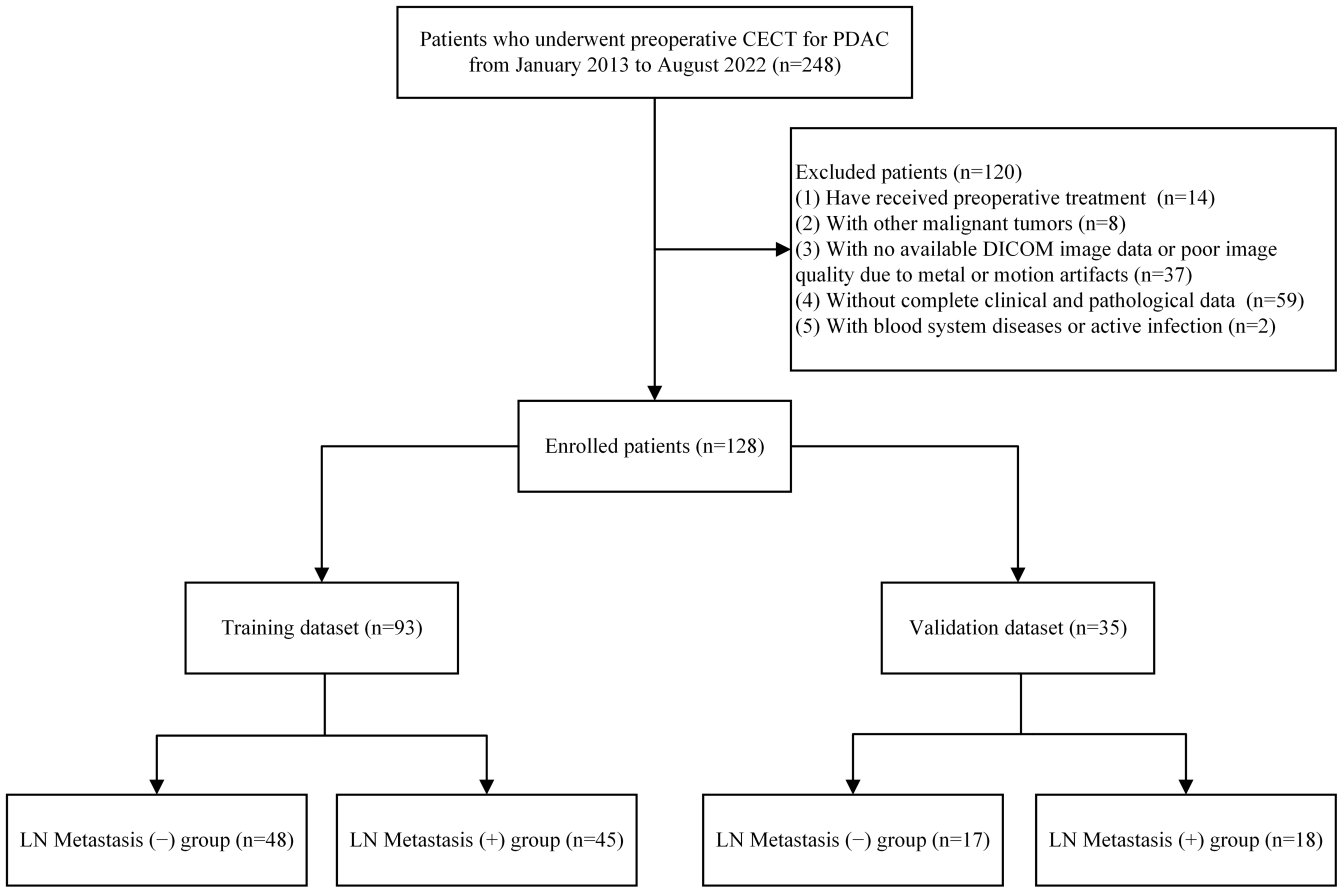
Flow chart of inclusion and exclusion criteria for patients.

This study was approved by the ethics committee of the First Affiliated Hospital of Fujian Medical University. This was a retrospective research of patients diagnosed with PDAC who underwent surgical resection with LN dissection treated at the First Affiliated Hospital of Fujian Medical University between January 2013 and August 2022. The inclusion criteria were: (1) patients who underwent surgical resection for pancreatic tumors and were pathologically confirmed with PDAC, and (2) patients who underwent multiphasic CECT of the pancreas within 1 month before surgery. The exclusion criteria were: (1) patients who underwent preoperative treatment, (2) patients with other malignant tumors, (3) patients with no available DICOM image data or poor image quality due to metal or motion artifacts, (4) patients without complete clinical and pathological data, and (5) patients with blood system diseases or active infection. A total of 128 patients met the inclusion and exclusion criteria and were included in the study. A total of 93 and 35 patients were selected at random for training and validation, respectively, with a ratio of 7:3. **Figure 1** illustrates the recruitment process and criteria for including and excluding patients.

### 2.2 Clinicopathological characteristics

The LN status was retrieved from the Pathology Information Management System. The clinical variables included age, gender, tumor size, body mass index (BMI), carcinoembryonic antigen (CEA), alpha-fetoprotein (AFP), carbohydrate antigen 125 (CA125), and carbohydrate antigen 199 (CA199). The neutrophil-to-lymphocyte ratio (NLR), derived neutrophil-to-lymphocyte ratio (dNLR), monocyte-to-lymphocyte ratio (MLR), systemic immune-inflammation index (SII), platelet-lymphocyte ratio (PLR), and prognostic nutritional index (PNI) were calculated as follows: NLR = neutrophil count/lymphocyte count, dNLR = neutrophil count/(white blood cell count - neutrophil count), PLR = platelet count/lymphocyte count, SII = platelet count * neutrophil count/lymphocyte count, MLR = monocyte count/lymphocyte count, PNI = albumin + 5 * lymphocyte count.

### 2.3 CT image acquisition

All patients diagnosed with PDAC were routinely subjected to CECT scans before undergoing surgery. Toshiba Aquilion PRIME 80-slice spiral CT or Toshiba Aquilion One 320-slice spiral CT scanner was utilized to obtain CT images using the following scanning parameters: tube voltage, 120 kV; tube current, 230 mAs; slice interval, 0 mm; slice thickness, 5 mm; rotation time, 0.35 s. The patients received contrast material intravenously through the ulnar vein using a high-pressure syringe with a flow rate of 3.0 mL/s prior to undergoing imaging. Subsequently, we acquired images corresponding to the arterial phase (AP), venous phase (VP), and equilibrium phase, with respective delays of 30, 60, and 120 s.

### 2.4 Tumor segmentation and feature extraction

**Figure 2:**
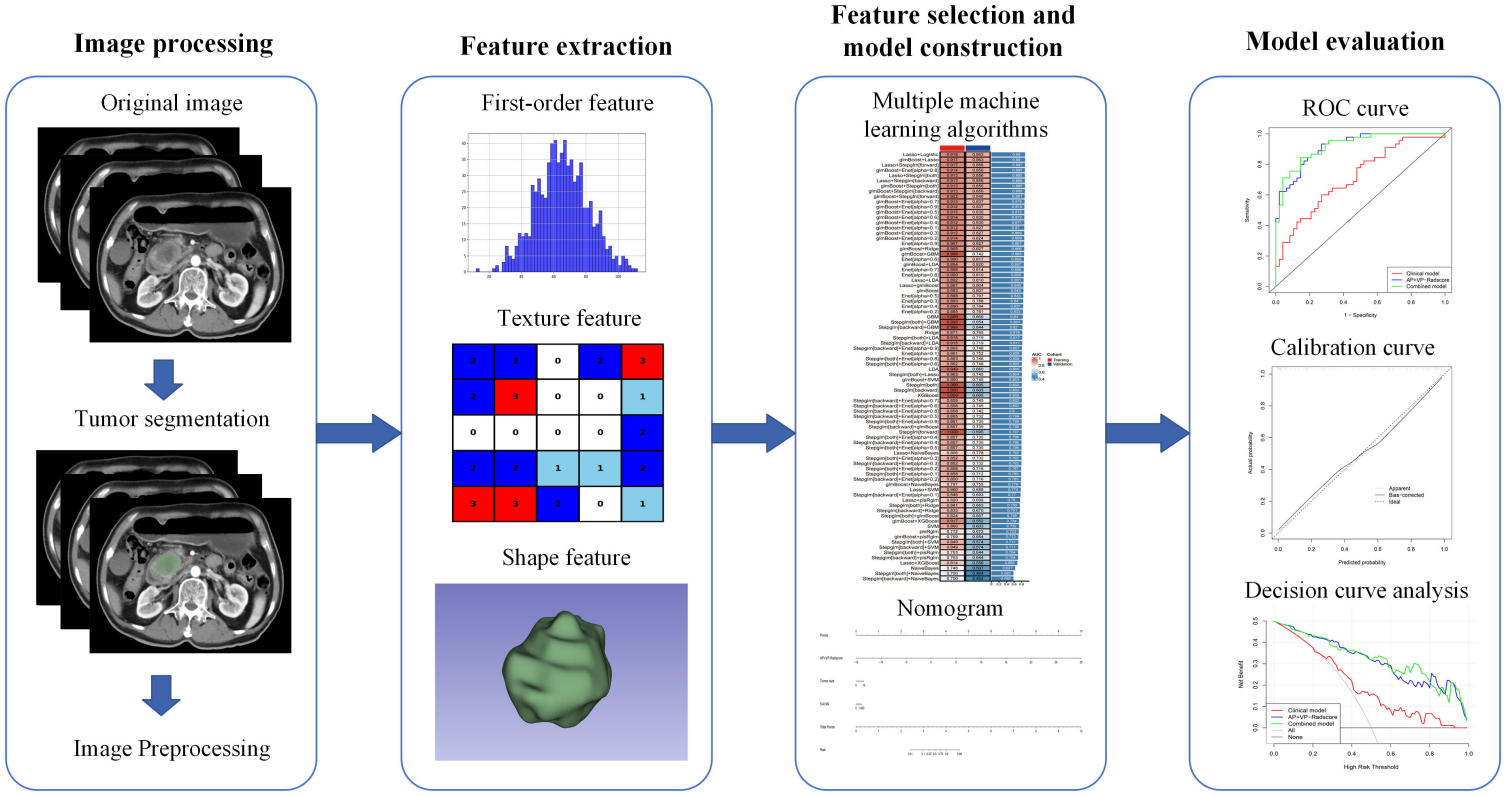
The workflow of model construction and validation.

**Figure 2** illustrates the research workflow. The CT pictures were acquired using the Picture Archiving and Communication System (PACS) implemented in the hospital. Applying 3D Slicer software (version 4.10.2), one radiologist (reader 1) manually segmented all regions of interest (ROIs) along the margin of the tumor from both the AP and VP images. To evaluate the repeatability of radiomics feature extraction, the CT images from 50 patients were randomly selected for ROI segmentation by reader 1 and a hepato-biliary-pancreatic surgeon (reader 2). The intraclass correlation coefficient (ICC) assessed the intra- and inter-observer agreements, with an ICC score above 0.75 indicating satisfactory agreement (15). For intra-observer reproducibility, reader 1 delineated the tumor ROIs twice within a month. To assess the inter-observer agreement, reader 2 independently delineated the ROI once and these results were compared to reader 1’s initial segmentation.

To achieve the voxel spacing standardization, the image data underwent resampling to a uniform size of 1.0 × 1.0 × 1.0 mm^3^. Additionally, image normalization was performed to mitigate variations in imaging among different CT scanning machines. The feature extraction procedure was based on the Image Biomarker Standardization Initiative (IBSI) and was implemented using the Pyradiomics package (version 3.0.1) in Python (version 3.7.4) (16). There were two distinct categories of radiomics features: original features and features derived by filter transformation. Those mentioned above included the shape, first-order, gray-level dependence matrix (GLDM), neighboring gray-tone difference matrix (NGTDM), gray-level co-occurrence matrix (GLCM), gray-level run length matrix (GLRLM), and gray-level size zone matrix (GLSZM). The latter incorporated wavelet and Laplace of Gaussian (LoG) filtering techniques. All features extracted from the image were normalized using z-score transformation.

### 2.5 Feature selection and radiomics model building

Two steps were taken for the selection of radiomics features in order to reduce overfitting or bias. First, relationships between the features and outcomes were examined in univariate analysis, and features with P-values lower than 0.05 were selected for further analysis. Second, to enhance the precision and reliability of the model, we incorporated a total of 13 distinct ML algorithms and explored 85 unique combinations of these methods (**Supplementary Table 1**). The integrative algorithms included ridge, least absolute shrinkage and selection operator (Lasso), elastic network (Enet), partial least squares regression for generalized linear models (plsRglm), linear discriminant analysis (LDA), generalized boosted regression modeling (GBM), random forest (RF), stepwise generalized linear model (Stepglm), support vector machine (SVM), boosted generalized linear model (glmboost), extreme gradient boosting (XGBoost), naive Bayes, and logistic regression. In the process, we used one algorithm to filter the variables and another algorithm to build the radiomics model. No hyper-parameter tuning of ML algorithms was performed, and default parameters were used. Radiomics models contained at least two features. The radiomics score (Radscore), which includes AP-Radscore, VP-Radscore, and AP+VP-Radscore, was respectively computed for each individual using the AP, VP, and AP+VP radiomics features in order to assess the model’s effectiveness. Finally, the area under the curve (AUC) was calculated for each model, and those exhibiting the highest mean AUC value was deemed the optimal model so that we could select robust and non-redundant features for preoperative prediction of the LN status of PDAC from the training cohort. The advantages and disadvantages of these ML algorithms can be found in the **Supplementary Material S1**.

### 2.6 Construction of the clinical and combined model

After univariate analysis comparing LN metastasis (–) and LN metastasis (+) patients’ demographic and laboratory data, clinical factors with P-values below 0.05 were retained for multivariate analysis. A clinical model was then constructed using clinical variables with P-values below 0.05 in the multivariate logistic regression (LR) analysis. Clinical model predictors and Radscore were used to construct a combined model by LR. The combined model was visually represented using a nomogram. Nomogram scores (Nomo-scores) were calculated using Radscore and significant clinical features.

### 2.7 Model evaluation

The validation datasets were used to assess the model’s performance. The performance of the model was assessed by AUC values calculated from the receiver operating characteristic (ROC) curve, along with 95% confidence interval (95%CI), sensitivity, specificity, accuracy, F1 score, recall, precision, positive predictive value (PPV) and negative predictive value (NPV) (17). Three different models, including the clinical model, Radscore, and the combined model, were evaluated and compared using the DeLong test. Five-fold cross-validation was applied to assess the model performance. The entire cohort was randomly divided into five similar-size subsamples. The model-building process was conducted on four subsamples and validated on the remaining one sub-sample. This process was repeated five times, ensuring each subsample was used once as the validation data. A calibration curve was used to evaluate the calibration of the model. Applying the Hosmer-Lemeshow test, the goodness-of-fit of the model was evaluated. In order to evaluate the effectiveness of the three models, a decision curve analysis (DCA) was performed by calculating the net benefit at different threshold probabilities.

### 2.8 Statistical analysis

The statistical analysis was carried out using SPSS (version 23.0) and R software (version 4.3.1). Continuous variables that followed a normal distribution were represented using the mean ± standard deviation, whereas those that did not adhere to normality were represented by the median (interquartile range). Numbers and percentages were used to represent categorical variables. Clinical and imaging features were analyzed for normality test by Shapiro-Wilk test and were analyzed for statistical differences by Student’s t-test, Mann-Whitney U test, and Chi-square test as appropriate. The “pROC” package of R software was used to construct ROC curves and calculate AUC. Calibration plots were constructed by the “rms” package. The DCA was constructed using the “rmda” package in the R program. Statistical significance was set at P-value < 0.05.

## 3 Results

### 3.1 Patient characteristics

A total of 128 patients were included in this study, including 80 males and 48 females. The patients were randomized into training (n=93) and validation (n=35) groups. **Table 1** summarizes the clinicopathological characteristics and demographics of all patients. The clinicopathologic variables in the training cohort did not differ significantly from those in the validation cohort.

**Table 1:** Demographic and clinicopathological characteristics of patients.

Characteristics	Training cohort (n =93)			Validation cohort (n =35)			P_inter_
	LN Metastasis (−)	LN Metastasis (+)	P_intra_	LN Metastasis (−)	LN Metastasis (+)	P_intra_	
Age	61.73 ± 11.72	61.49 ± 9.94	0.916	60.94 ± 10.24	63.28 ± 9.86	0.496	0.802
BMI	22.02 ± 2.74	21.49 ± 3.16	0.390	21.29 ± 2.11	21.65 ± 3.41	0.715	0.621
Gender			0.050			0.407	0.720
Male	35(72.92)	24(53.33)		9(52.94)	12(66.67)		
Female	13(27.08)	21(46.67)		8(47.06)	6(33.33)		
HBP			0.184			0.088	0.979
No	36(75.00)	28(62.22)		14(82.35)	10(55.56)		
Yes	12(25.00)	17(37.78)		3(17.65)	8(44.44)		
Diabetes			0.805			0.193	0.645
No	30(62.50)	27(60.00)		13(76.47)	10(55.56)		
Yes	18(37.50)	18(40.00)		4(23.53)	8(44.44)		
CA125	15.88(10.68-21.70)	22.30(13.75-51.00)	0.012	25.32(10.95-40.75)	20.54(9.49-39.98)	0.843	0.728
CA199	118.50(22.43-239.70)	244.00(95.45-812.95)	0.006	28.20(7.19-236.40)	258.00(83.23-777.30)	0.045	0.561
CEA	4.18(2.46-6.35)	2.84(2.05-5.38)	0.087	3.25(2.26-5.84)	3.39(2.16-6.22)	0.961	0.472
AFP	2.14(1.61-3.00)	2.43(1.71-3.38)	0.254	2.76(1.86-4.82)	2.54(1.74-3.19)	0.400	0.281
Tumor size	3.30(2.50-4.48)	4.50(2.85-5.85)	0.003	3.50(2.80-5.15)	3.65(3.10-5.75)	0.418	0.738
LYM	1.58 ± 0.58	1.48 ± 0.50	0.360	1.49 ± 0.59	1.39 ± 0.55	0.594	0.391
WBC	6.26(5.17-7.33)	5.46(4.67-6.77)	0.220	6.07(4.92-6.82)	5.75(4.21-6.76)	0.488	0.485
NEU	3.67(2.69-4.61)	3.61(2.84-4.52)	1.000	3.42(2.96-4.28)	3.02(2.42-4.53)	0.338	0.499
M	0.36(0.30-0.45)	0.31(0.26-0.40)	0.100	0.36(0.29-0.40)	0.33(0.30-0.50)	0.608	0.634
PLT	226.00(196.25-266.50)	212.00(166.50-258.00)	0.214	211.00(176.00-293.50)	210.50(159.75-265.50)	0.563	0.441
NLR	2.40(1.73-3.41)	2.73(1.80-3.60)	0.299	2.48(1.93-3.13)	2.56(1.75-2.95)	0.741	0.779
dNLR	1.64(1.20-2.25)	1.90(1.34-2.31)	0.155	1.68(1.39-2.11)	1.69(1.22-1.99)	0.509	0.598
PLR	158.71(110.76-195.24)	142.74(118.35-204.69)	0.939	144.05(122.29-199.19)	156.66(116.66-195.32)	1.000	0.730
SII	536.45(367.49-849.28)	531.10(388.12-796.42)	0.945	612.86(327.41-808.38)	437.38(323.53-598.28)	0.448	0.427
MLR	0.23(0.19-0.33)	0.22(0.17-0.35)	0.628	0.21(0.19-0.32)	0.27(0.17-0.39)	0.597	0.532
PNI	48.24 ± 5.38	48.15 ± 5.69	0.939	47.19 ± 5.53	47.24 ± 5.41	0.977	0.368

HBP, high blood pressure; NEU, neutrophil; LYM, lymphocyte; WBC, white blood cell; M, monocyte; PLT, platelet; P_intra_ is the result of univariate analyses between the LN metastasis (−) and LN metastasis (+) groups while P_inter_ represents whether a significant difference exists between the training and validation datasets.

### 3.2 Inter- and intra-observer agreements

There were 1316 features extracted from AP and VP images. Among the 1316 AP radiomic features, the satisfactory consistency rate of features of intra-observer agreement was rated at 79.8% (mean ICC= 0.841, **Supplementary Figure 1A**), and inter-observer agreement was 78.3% (mean ICC= 0.823, **Supplementary Figure 1B**). For the 1316 VP radiomic features, the satisfactory consistency rate of features of intra-observer agreement was rated at 79.0% (mean ICC= 0.831, **Supplementary Figure 1C**), and inter-observer agreement was 79.4% (mean ICC= 0.828, **Supplementary Figure 1D**). Of the 2632 AP+VP radiomic features, the satisfactory consistency rate of features of intra-observer agreement was rated at 79.4% (mean ICC= 0.836, **Supplementary Figure 1E**), and inter-observer agreement was 78.8% (mean ICC= 0.825, **Supplementary Figure 1F**). A total of 989 AP, 977 VP, and 1966 AP+VP radiomic features had intra- and inter-observer ICCs greater than 0.75, indicating good reproducibility.

### 3.3 Feature selection and radiomics model building

**Figure 3:**
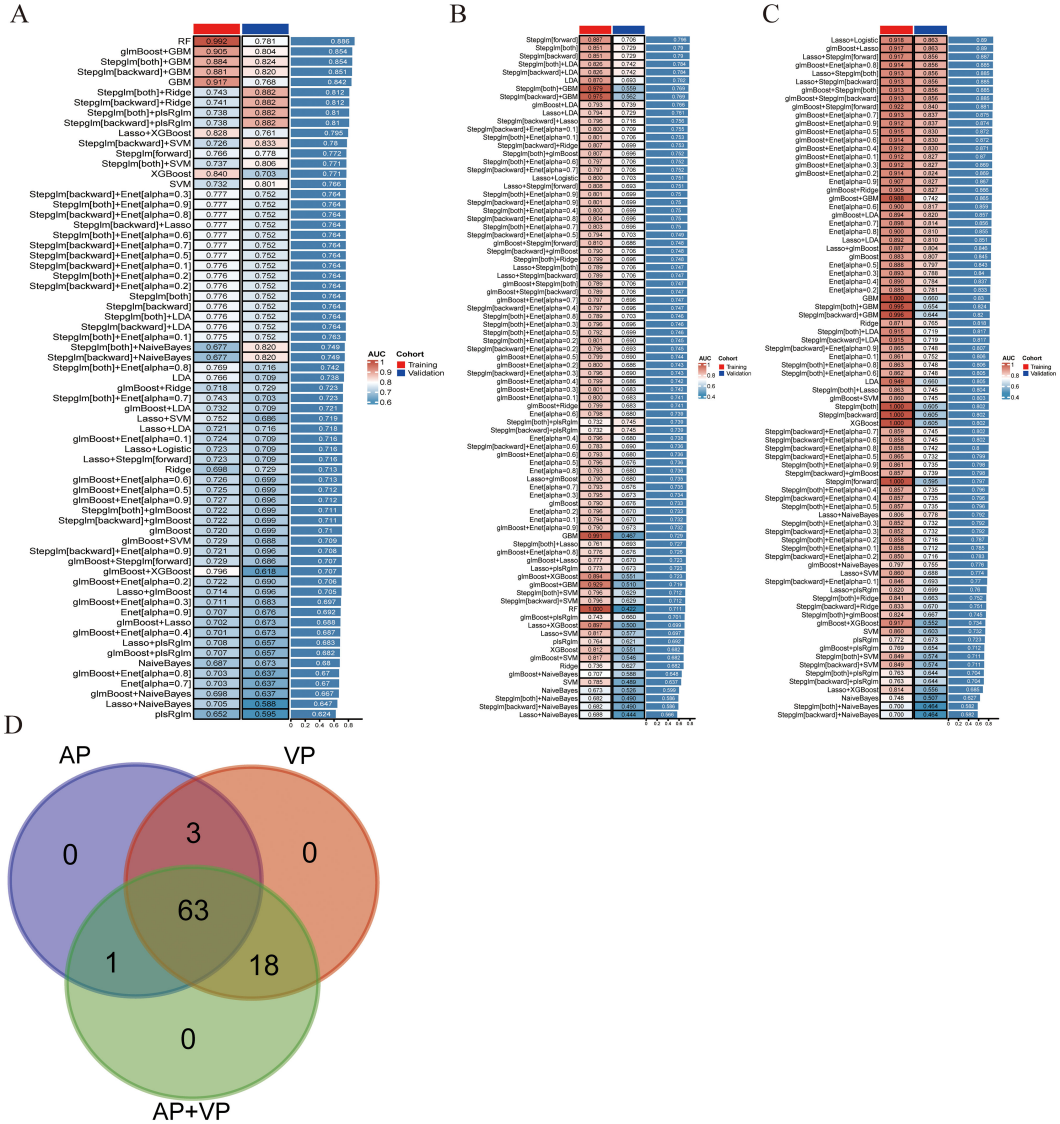
Develop and validate models through integrated machine learning. **(A)** A total of 67 prediction models were built using features from arterial phase radiomics, and the AUC of each model was further computed on the training and validation datasets. **(B)** 84 predictive models built from venous phase radiomics features. **(C)** 82 predictive models constructed based on radiomics features of arterial and venous phases. **(D)** Wayne diagram of the number of models.

**Figure 4:**
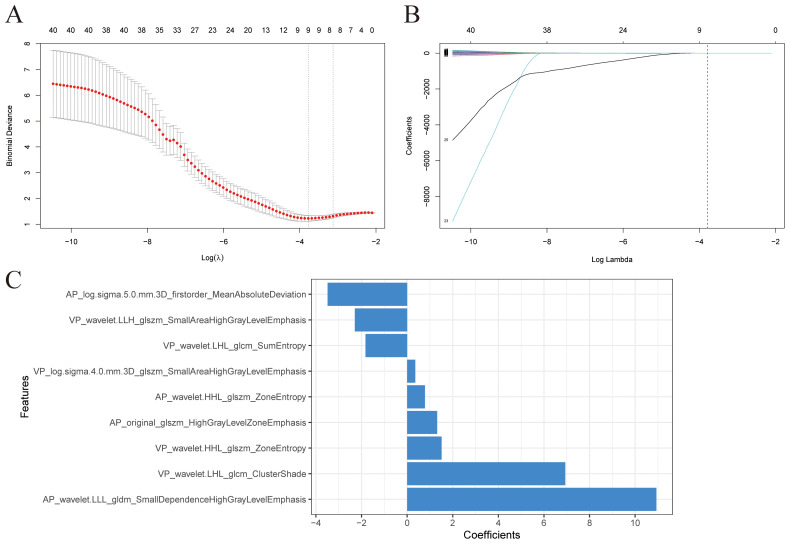
Radiomics features selection. **(A)** The value (λ) was chosen for the Lasso algorithm’s tuning parameter through 10-fold cross-validation. Two vertical lines show the optimal values according to the minimum criterion and 1-SE criterion. The optimal λ value of 0.0229 was chosen. **(B)** Profiles of Lasso coefficients for 41 radiomics features. A vertical line was drawn at the value selected using 10-fold cross-validation, and a total of nine features with non-zero coefficients were chosen. **(C)** Nine selected radiomics features and their coefficients.

After univariate analysis, 12, 29, and 41 features were retained for AP, VP, and AP+VP, respectively. Subsequently, different algorithm combinations were used to construct 67 AP, 84 VP, and 82 AP+VP models. Among the models constructed with AP features, the RF model had the best predictive efficacy, achieving AUCs of 0.992 and 0.781 for the training and validation groups, respectively (**Figure 3A**). The Stepglm (direction = forward) model had the best predictive efficacy among the models constructed with VP features, with AUCs of 0.887 and 0.706 for the training and validation groups, respectively (**Figure 3B**). Among the models that were built using AP+VP features, it was observed that the Lasso+Logistic model exhibited the highest effectiveness in predicting the status of LNs. The AUCs for this model were determined to be 0.918 and 0.863 for the training and validation cohorts, respectively (**Figure 3C**). The Wayne diagram of the number of models constructed from the three-period phases is shown in **Figure 3D**. Among the models constructed using AP, VP, and AP+VP features, the Lasso+Logistic model using AP+VP features demonstrated the best predictive performance. Therefore, further analysis was focused on this model. In the Lasso+Logistic model using AP+VP features, Lasso selected nine features with non-zero coefficients (**Figures 4A, B**). Furthermore, the Lasso+Logistic model using AP+VP features was quantitatively integrated into AP+VP-Radscore. The equation for computing the radiomics score is presented in the **Supplementary Material S2**. **Figure 4C** shows the detailed feature names and their corresponding coefficients.

### 3.4 Clinical model and combined model construction

Univariate analysis indicated that tumor size, CA199 and CA125 levels were statistically significant (P<0.05). Multivariate analysis indicated that CA199 level (OR=1.001 [1.000–1.003], P=0.043) and tumor size (OR=1.379 [1.079–1.763], P=0.010) were independent predictors of PDAC LN status (**Table 2**). These two variables developed a clinical model. A combined model was constructed by integrating clinical variables, including CA199 and tumor size, with the AP+VP-Radscore.

**Table 2:** Clinical factors in the multivariate analysis.

Characteristics	Coefficient	P value	OR	95% CI of OR
CA199	0.001	0.043	1.001	(1.000, 1.003)
CA125	0.003	0.450	1.003	(0.995, 1.011)
Tumor size	0.321	0.010	1.379	(1.079, 1.763)
Constant	-1.930	0.001	0.145	-

OR, odds ratio.

### 3.5 Model evaluation

**Figure 5:**
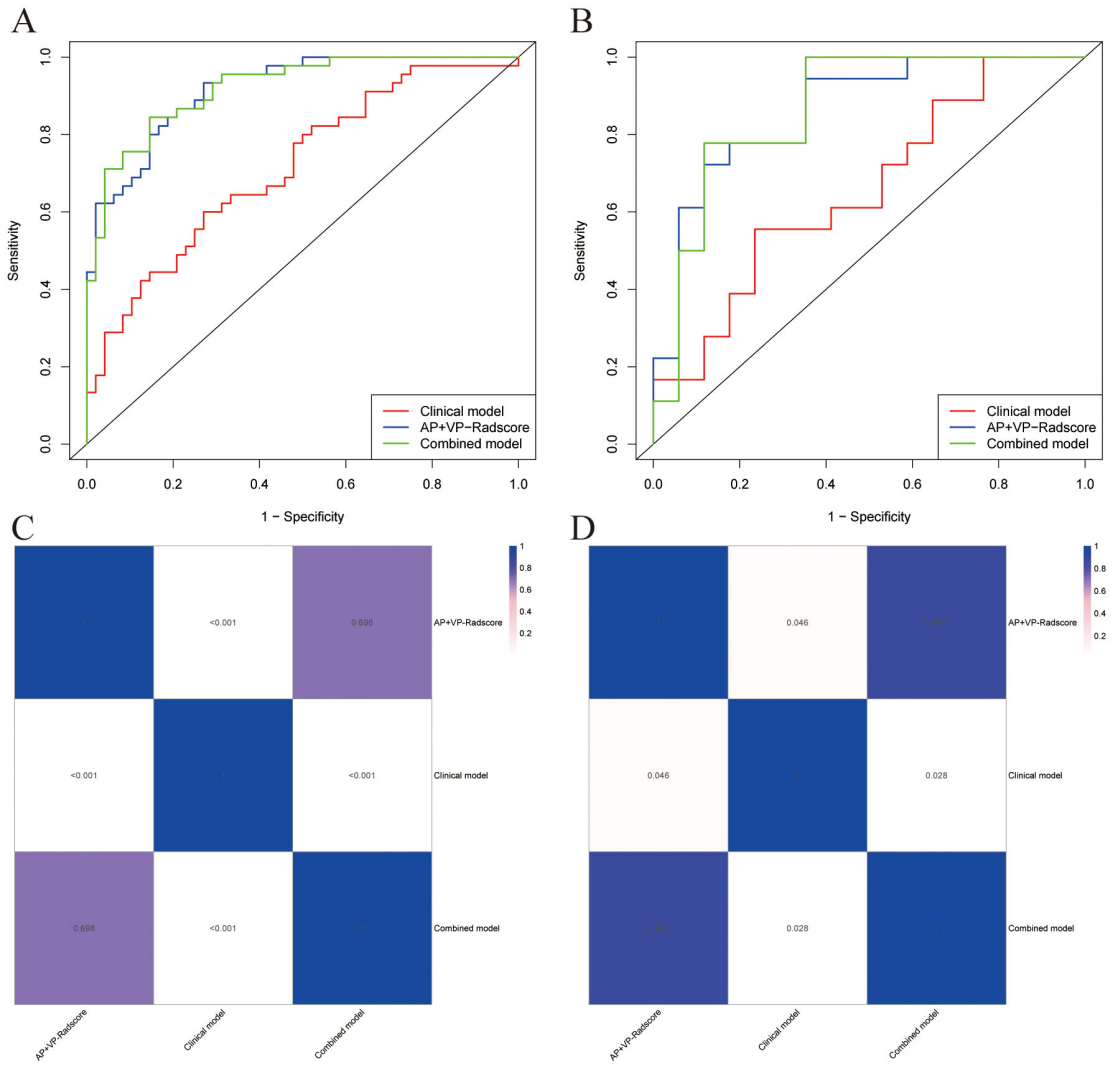
The comparative predictive performance of various models. ROC curves of different models in the training **(A)** and validation **(B)** cohorts; Heat map of the P-values for the DeLong test between the training **(C)** and validation **(D)** cohorts of different models.

We built a clinical model, AP+VP-Radscore, as well as a combined model. A comparison of the ROC curves for all prediction models can be seen in **Figures 5A, B**. The clinical model exhibited suboptimal performance, as evidenced by the AUC values of 0.714 and 0.657 in the training and validation datasets, respectively. The combined model had superior predictive performance compared to the other two models. This model exhibited sensitivity, specificity, and AUC values of 84.44%, 85.42%, and 0.920 (95% CI: 0.868-0.973), respectively, in the training dataset, and 77.78%, 88.24%, and 0.866 (95% CI: 0.737-0.995), respectively, in the validation dataset (**Table 3**). The DeLong test presented that the combined model and AP+VP-Radscore exhibited significantly enhanced predictive performances (AUCs: 0.920 vs 0.714, P <0.001 and AUCs: 0.918 vs 0.714, P <0.001, respectively) compared to the clinical model in the training set (**Figures 5C, D**). Additionally, the combined model demonstrated higher prediction accuracy in comparison to the AP+VP-Radscore; however, this improvement did not reach a statistical significance in either the training or validation datasets. Among all the three models, the combined model outperformed the other two models, whether in training or validation datasets. Subsequently, the performance of the models was evaluated using five-fold cross-validation (**Supplementary Table 2**). The combined model demonstrated stable prediction performance in both the training (mean AUC± standard deviation [SD], 0.909 ± 0.009) and validation (mean AUC ± SD, 0.904 ± 0.037) cohorts.

**Table 3:** Predictive performance of different models.

Models	Training cohort (n = 93)									Validation cohort (n = 35)								
	AUC (95% *CI*)	Sensitivity (%)	Specificity (%)	Accuracy (%)	F1 score (%)	Recall (%)	Precision (%)	PPV (%)	NPV (%)	AUC (95% *CI*)	Sensitivity (%)	Specificity (%)	Accuracy (%)	F1 score (%)	Recall (%)	Precision (%)	PPV (%)	NPV (%)
Clinical model	0.714(0.610-0.818)	60.00	72.92	66.67	63.53	60.00	67.50	67.50	66.04	0.657(0.472-0.842)	55.56	76.47	65.71	62.50	55.56	71.43	71.43	61.90
AP+VP-Radscore	0.918(0.865-0.970)	93.33	72.92	82.80	84.00	93.33	76.36	76.36	92.11	0.863(0.739-0.987)	72.22	88.24	0.800	78.79	72.22	86.67	86.67	75.00
Combined model	0.920(0.868-0.973)	84.44	85.42	84.95	84.44	84.44	84.44	84.44	85.42	0.866(0.737-0.995)	77.78	88.24	82.86	82.35	77.78	87.50	87.50	78.95

**Figure 6:**
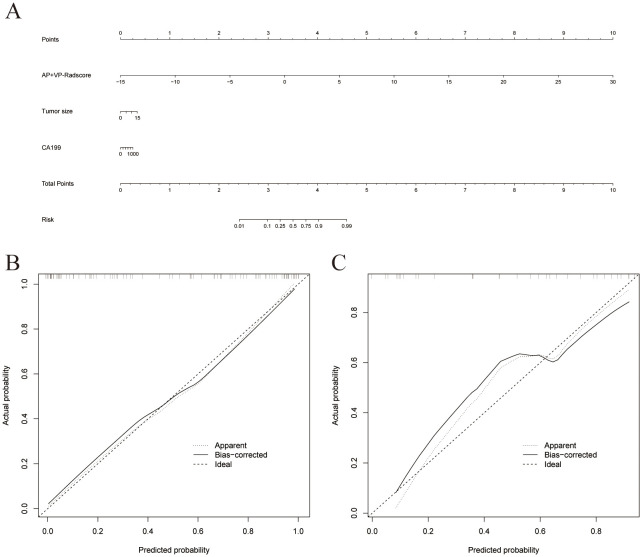
**(A)** A nomogram was plotted by combining AP+VP-Radscore with independent clinical predictors in the training cohort. Nomogram calibration curves for the training **(B)** and validation **(C)** cohorts.

**Figure 7:**
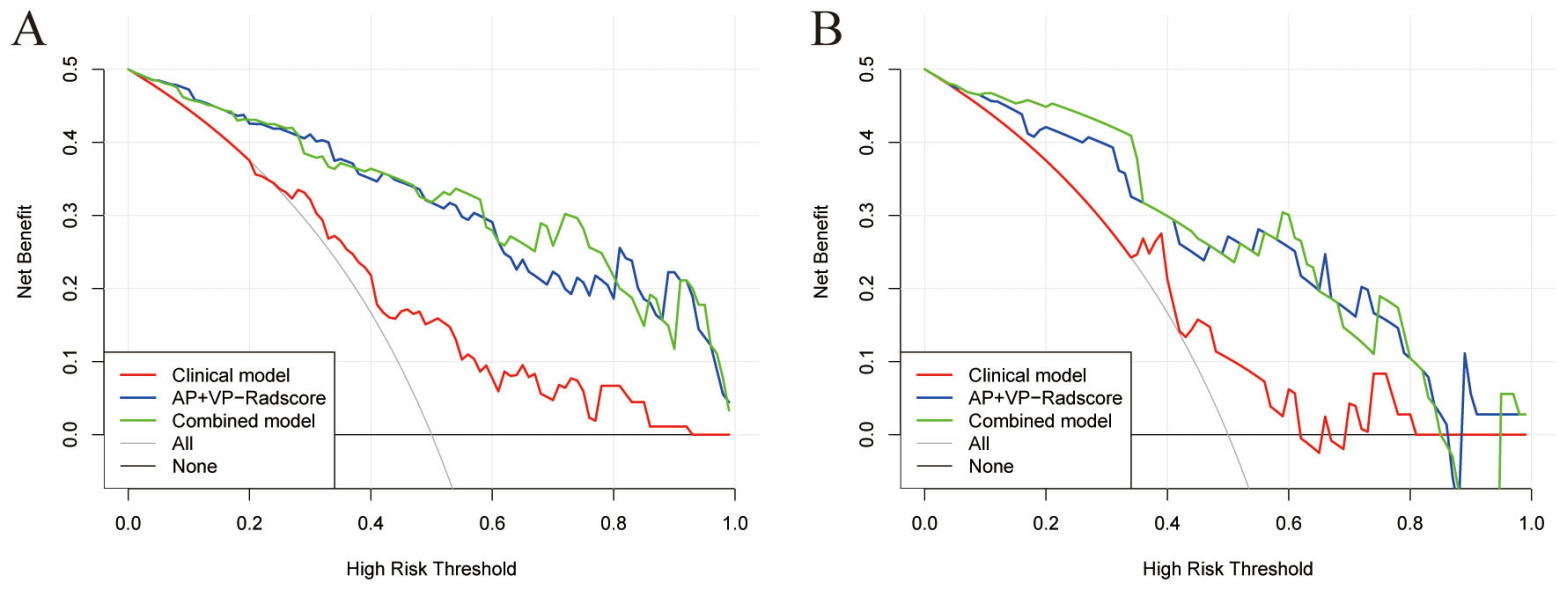
DCA curves for three models in the training **(A)** and validation sets **(B)**.

To determine the LN status of PDAC, we developed a nomogram based on the combined model (**Figure 6A**). **Supplementary Figure 2** displays the Nomo-scores of every individual in both the training and validation groups. **Supplementary Figure 3** shows the CECT images and hematoxylin-eosin stained pictures with and without LN metastasis. Nomogram calibration curves demonstrated favorable calibration for training and validation groups (**Figure 6B, C**). The Hosmer-Lemeshow test showed no significant difference between the predicted calibration and ideal curves in the training (χ^2^ = 3.705, P=0.883) and validation (χ^2^ = 13.654, P=0.091) cohorts. This suggests that the nomogram exhibited a good level of conformity in both the cohorts. **Figures 7A, B** displays the DCA curves of the three models. The combined model exhibited superior net benefit in accurately identifying the LN status of PDAC in both the training and validation populations.

## 4 Discussion

PDAC has a heightened mortality rate and a dismal prognosis, mostly attributed to the challenges associated with early identification and the curative treatment modalities. In patients with PDAC, pancreatectomy has the greatest likelihood of improving long-term survival. LN status as an essential and independent prognostic indicator has been demonstrated (18). However, there remains a debate as to whether standard or extended LN dissection should be included during pancreatectomy (19, 20). Therefore, it is essential to accurately stage the LN before surgery in order to provide patients with comprehensive counseling on surgical choices and prognosis. The objective of this research was to construct and verify multiple ML models using CECT to differentiate the LN status in PDAC. As the combined model incorporated elements like CA199 levels and tumor size, it varied from the conventional single radiomics model. The combined model demonstrated significant capability of distinguishing LN metastasis in the training (AUC, 0.920) and validation (AUC, 0.866) cohorts. To the best of our knowledge, this is the first study to compare multiple ML algorithms to detect the LN metastases in PDAC preoperatively.

CA199 is currently regarded as the most appropriate serum marker for patients with resectable PDAC, as it is the only prognostic biomarker approved by the Food and Drug Administration (21). A study has provided evidence of the clinical significance of the preoperative CA199 level as a prognostic marker in patients with PDAC presenting with metastases in the para-aortic LN (22). In our study, the CA199 level was validated as an independent risk factor of LN metastasis for PDAC. We speculated that such results may be because CA199 is a particular biomarker that reflects the biological activity and that the greater the CA199 level, the worse the patient’s prognosis (23). A study found a positive association between the size of tumors and occurrence of LN metastases in endometrial carcinoma (24). Similarly, our investigation has unveiled that the size of the tumor serves as a noteworthy and independent predictor of the occurrence of LN metastasis. However, clinical models consisting of CA199 and tumor size have poor predictive ability for LN status (AUC= 0.714 in training datasets; AUC = 0.657 in validation datasets). Some studies suggested that patients with severe obstructive jaundice may have elevated CA199, which can mislead predictive results (25). It partially explains the poor predictive efficacy of clinical models. The predictive value of inflammatory indices in relation to the prognosis of individuals diagnosed with PDAC has been shown in previous studies (26, 27). However, our investigation could not confirm the association between inflammatory indices and LN metastases. The differences in the study population may explain the inconsistencies with previous findings, and research with larger sample sizes or in other populations is warranted in the future.

Although CT is the most commonly used method to evaluate pancreatic cancer resectability, Imai et al. (28) indicated that low-order data such as LN diameter and volume measured from CT image cannot reflect obvious differences between patients with pancreatic cancer with and without LN metastasis. This may explain why visual assessment of CT scans has a low efficiency for detecting LN metastases among patients with pancreatic cancer. Radiomics, also referred to as a “whole-tumor virtual biopsy technique,” enables the extraction of numerous image features from the entire tumor, reflecting its heterogeneity and characteristics (29). A biopsy examines only a portion of the tumor tissue, which cannot comprehensively assess the intra-tumor heterogeneity before surgery (30). In contrast, radiomics can non-invasively reflect comprehensive information about the entire tumor. This approach has promise for enhancing diagnostic capabilities and facilitating the development of personalized treatment approaches (31). The data from radiomics contain first-, second-, and higher-order features that can hardly be identified by the naked eye (31). This data was used for predicting the LN status of bladder, breast, and gastric, and esophageal cancers and were suggested to have some advantages (32–35). Several studies in recent years have used CECT radiomics analysis of primary lesions to predict LN metastasis. PDAC tumors or peri-tumors have been shown to have a high lymphatic vessel density (36, 37). PDAC commonly infiltrates the LNs through the lymphatic system, as opposed to direct or adjacent extension of the primary cancer into the LN (38, 39). Accordingly, analyzing the image of the primary tumor may be more effective than the image of LNs in predicting LN metastasis. That is why ROIs were segmented into tumor regions in this study. Some previous studies have used only one VP radiomics feature to construct the models; however, our study shows that AP radiomics feature is also important (12, 40, 41). Some other studies have only considered AP images, which may miss some useful information about the VP images (7, 42). Our findings suggest that AP and VP radiomics feature have comparable performances in predicting the LN status. Besides, the predictive performance of the AP+VP-Radscore constructed by combining AP and VP features was higher than that of the model constructed by a single phase. Thus, incorporating more CT phases may allow for the development of more effective models. Shi et al. conducted a study wherein they employed MRI radiomics to detect LN metastasis in individuals with PDAC prior to surgery (43). However, it should be noted that their study incorporated a limited number of clinical factors. A previous study developed a radiomics-based strategy for preoperative prediction of LN status in patients with resectable PDAC. However, only the largest tumor strata were examined in their investigation, and the sample comprised only 85 cases (41). Most of these previous studies used only one algorithm, hence limited conclusions can be drawn. Our study differed from previous studies as we incorporated AP and VP of CT rather than one scan phase. Multiple radiomics ML models were compared rather than solely focusing on a single model. The integration of the clinical variables and the AP+VP-Radscore in our combined model demonstrated a high level of predictive capability, as evidenced by an AUC of 0.866 in the validation cohort. This performance surpassed that of both the single phase Radscore and clinical model. Hence, the inclusion of clinical variables holds significance and warrants consideration in the analysis of radiomics.

In this study, the predictive power of the combined AP and VP features outperformed that of separate AP or VP model. The AP+VP-Radscore in our study was calculated by six wavelet texture, two LoG filtered texture, and one original features. Wavelet features have been confirmed as useful indexes in predicting the tumor pathological grade and histologic subtype (44, 45). According to our study, the wavelet texture features took an important position in the LN metastasis prediction model. By using the wavelet decomposition of images at several scales, fine and coarse textures could be extracted to depict the spatial heterogeneity within tumor regions (46). The findings of this research align with those of other studies that employed wavelet-based features in their radiomics models. A previous research endeavor was initiated to construct a predictive model to assess the preoperative LN status for intrahepatic cholangiocarcinoma, which was constructed using wavelet features exclusively (47). In another research aimed at evaluating the role of radiomics in predicting LN status in intrahepatic cholangiocarcinoma, it was observed that wavelet features constituted 79% of the features incorporated into the model (48). Xiao et al. conducted research to establish and validate a nomogram for the purpose of forecasting the occurrence of LN metastasis in individuals with early-stage cervical cancer. The constructed model encompassed a total of 23 features, with 19 of these being wavelet features (49). We speculated that the possible explanation for this phenomenon could be the potential correlation between wavelet features and the infiltration of malignancies into the lymphatic system. LoG filtering is a sophisticated technique for picture filtering that integrates the principles of Laplace and Gaussian filtering. The utilization of Laplacian filtering in image processing can effectively emphasize locations within the image that have undergone mutations in gray level, hence enhancing the contrast in gray levels. The application of a Gaussian operator can mitigate the noise introduced by the Laplace operator (50, 51). LoG filtering can also be beneficial in detecting LN metastases of PDAC based on the results of our study. Our results show that GLSZM features are helpful in diagnosing metastatic LNs in PDAC. A previous study used radiomics to predict LN metastasis in individuals diagnosed with PDAC, and the model also included GLSZM features (12). GLSZM features quantify gray level zones in an image. The texture features based on GLSZM, which capture the interplay of adjacent pixels, have demonstrated a superior ability to quantify tumor texture and heterogeneity compared to features based on histograms (52).

ML algorithms, including SVM, LDA, and LR, have been extensively employed in the domain of clinical prediction and classification tasks (53, 54). Multiple studies show that LR has better performance than other ML algorithms (55–58). A meta-analysis which compared the efficacy of multiple ML algorithms in pregnancy care prognosis prediction indicated that LR models had superior performance compared to non-LR models in trials with a low risk of bias (57). Li et al. (58) employed stepwise selection using LR as well as four ML techniques, including gradient boosting machine, RF, XGBoost, and single-layer neural networks, in order to construct predictive models for hip fractures. LR was found to be best in terms of discrimination performance. ML has demonstrated high performance in tasks characterized by a significant signal-to-noise ratio, such as identification of handwriting and video games. Clinical predictive issues frequently exhibit one low signal-to-noise ratio (59). LR is based on a sigmoid function and works best on binary classification problems (60). Consequently, LR with universality cannot be ignored in this study. To explore the best algorithm for predicting PDAC LN status, we compared 233 models and finally confirmed that the Lasso-Logistic regression model constructed with AP+VP features outperformed other ML algorithms. This discovery implies that the efficacy of the model is impacted by the characteristics of the algorithm and its congruence with the study goals.

Our study had several limitations. First, it was only based on single-center data. Although our study adopted cross-validation, it may not avoid the overfitting risk. A large sample, multicenter study utilizing multiple CT scanners is necessary to validate the accuracy and stability of our combined model. Second, subgroup analysis based on sites of LN metastases was not implemented. Third, we only evaluated the AP and VP images of CECT; combining the plain and delayed phases may contain more information about tumor heterogeneity. Moreover, this study did not employ dynamic radiomics features to quantify the degree of variation between AP and VP, thereby overlooking the temporal evolution of radiomics features. Dynamic radiomics features should also be explored in future studies. Fourth, we used the default parameter settings of ML algorithms instead of tuning the parameters, which may have hindered the algorithm from achieving its optimal performance. Finally, the image segmentation approach utilized in this study relied on manual delineation. In the future, automated segmentation techniques could be employed to enhance consistency and efficiency.

In summary, of the 233 radiomics models examined, the model built by applying AP+VP radiomics features and a combination of Lasso-Logistic algorithm had the most favorable performance in both the training and validation cohorts. Our investigation showed that integrating the AP+VP-Radscore with clinical parameters yielded the best performance. This combined model has the potential to serve as an accurate and non-invasive instrument for forecasting LN metastasis of PDAC, hence facilitating clinical decision-making.

## Data Availability

The original contributions presented in the study are included in the article/Supplementary Material, further inquiries can be directed to the corresponding author/s.
