# Supplementary material for: Contrast-enhanced CT radiomics combined with multiple machine learning algorithms for preoperative identification of lymph node metastasis in pancreatic ductal adenocarcinoma
Source: Front Oncol. 2024 Sep 13;14:1342317. doi: 10.3389/fonc.2024.1342317 (PMC11427235; doi:10.3389/fonc.2024.1342317)
Supplement: Supplementary file 1 [file DataSheet1.docx]

Supplementary Material

# Supplementary Data

**Supplementary Material S1.** The advantages and disadvantages of the machine learning algorithms used in this study.

- 1. **1. Ridge Regression**

**Selection reason:** Ridge regression is an analytical method to address multicollinearity. Ridge regression employs a shrinkage strategy by adding an L2 regularization term to the loss function, effectively reducing model coefficients and preventing overfitting. This is particularly important in high-dimensional datasets like radiomics data, which often have many features and are prone to overfitting. Ridge regression's regularization can mitigate the high collinearity among radiomics features, improving model stability and predictive performance. As a linear model, Ridge regression offers good interpretability, aiding clinicians in understanding the influence of each feature on the predictive outcome, which is crucial for clinical applicability.

**Advantages:**

- Prevents overfitting: Using L2 regularization, Ridge regression effectively reduces overfitting, leading to better generalization on training and test data.
- Handles high-dimensional data: Ridge regression works well when the number of features exceeds the number of samples, making it suitable for radiomics datasets.
- Addresses multicollinearity: Ridge regression reduces the impact of multicollinearity among features, enhancing model stability and accuracy.
- Interpretability: As a linear model, Ridge regression clearly shows the coefficient of each feature, aiding researchers and clinicians in understanding the model's decision process.

**Disadvantages:**

- Inability to capture non-linear relationships: Being a linear model, Ridge regression may not adequately capture complex non-linear relationships in the data.
- Hyperparameter selection: Choosing the appropriate regularization parameter (alpha) is crucial and typically requires cross-validation.
- Need for feature scaling: Ridge regression is sensitive to the scale of features, necessitating standardization before training.
  1. **2. Lasso Regression**

**Selection reason:** Lasso Regression uses L1 regularization, which can shrink some feature coefficients to zero, thus performing feature selection. This is particularly useful in high-dimensional data as it reduces the number of features, enhancing model interpretability and stability. In CT radiomics data, Lasso regression can simplify the model structure and improve predictive performance, although it may lose some information when handling correlated features.

**Advantages:**

- Feature selection: By shrinking some coefficients to zero, Lasso regression effectively selects the most relevant features, simplifying the model and improving interpretability.
- Reduces overfitting: The regularization term reduces model complexity, lowering the risk of overfitting.

**Disadvantages:**

- Information loss: When dealing with highly correlated features, Lasso regression may select only one feature, ignoring other useful ones.
- Hyperparameter selection: The regularization parameter needs to be chosen carefully, often requiring cross-validation.
  1. **3. Elastic Network (Enet)**

**Selection reason:** Elastic network combines the advantages of L1 and L2 regularization, making it suitable for high-dimensional data and multicollinearity issues while enabling feature selection.

**Advantages:**

- Effectively handles multicollinearity: Combines L1 and L2 regularization to address multicollinearity.
- Feature selection: Capable of selecting features to simplify the model.

**Disadvantages:**

- Hyperparameter tuning: Requires determination of regularization parameters, often needing cross-validation.
  1. **4. glmBoost**

**Selection reason:** glmBoost is based on gradient boosting, iteratively selecting and weighting features to achieve feature selection. It can handle complex data relationships, making it suitable for high-dimensional data. In CT radiomics data, glmBoost improves predictive accuracy through iterative optimization but requires careful parameter tuning.

**Advantages:**

- Feature selection: Iterative optimization selects the most relevant features, enhancing model performance.
- Handles complex relationships: Suitable for high-dimensional data with complex relationships.

**Disadvantages:**

- Poor interpretability: The complexity of the model makes it difficult to understand its internal mechanisms.
- Complex parameter tuning: Requires careful parameter adjustment to avoid overfitting.
  1. **5. Stepwise Generalized Linear Model**

**Selection reason:** Stepwise regression incrementally selects and adds features to build the optimal model. It reduces model complexity and enhances interpretability through stepwise selection and exclusion of features.

**Advantages:**

- Feature selection: Reduces model complexity and enhances interpretability.
- Easy to understand: The stepwise building process is intuitive and easy to explain.

**Disadvantages:**

- May overlook important features: Important features that interact with other variables may be missed.
- Long computation time: Stepwise regression can be time-consuming, especially with high-dimensional data.
  1. **6. Support Vector Machine (SVM)**

**Selection reason:** SVM uses kernel functions to handle high-dimensional feature spaces and is suitable for non-linear classification problems. It performs well with small sample sizes, making it suitable for CT radiomics data classification tasks.

**Advantages:**

- Handles non-linearity: Kernel functions effectively address non-linear classification problems and are suitable for high-dimensional data.
- Small sample advantage: Provides good generalization with small sample sizes.

**Disadvantages:**

- Parameter sensitivity: Sensitive to parameter choices (e.g., kernel type and regularization parameter), making parameter tuning complex.
- Computational complexity: Training time is long, especially with large datasets.
  1. **7. Extreme Gradient Boosting (XGBoost)**

**Selection reason:** XGBoost enhances gradient boosting tree models, offering efficient computation and strong predictive performance. It excels in handling missing values and complex data relationships, making it suitable for CT radiomics data classification.

**Advantages:**

- Efficient computation: High computational efficiency and strong predictive performance, capable of handling complex data relationships.
- Prevents overfitting: Built-in cross-validation and early stopping mechanisms effectively prevent overfitting.

**Disadvantages:**

- Complex parameter tuning: Requires significant computational resources for parameter optimization.
- Overfitting risk: Despite having mechanisms to prevent overfitting, it can still occur in some cases.
  1. **8. Random Forest (RF)**

**Selection reason:** RF constructs multiple decision trees and aggregates their results, making it suitable for high-dimensional data. It performs well with multicollinearity, making it appropriate for CT radiomics data classification.

**Advantages:**

- Robustness: Handles high-dimensional and multicollinear data well and is insensitive to outliers.
- Feature Selection: Capable of selecting features and handling missing values.

**Disadvantages:**

- High model complexity: Training time is long, especially with large datasets, leading to high computational complexity.
- Poor Interpretability: Explaining the model's decision process is difficult, exhibiting strong black-box characteristics.
  1. **9. Naive Bayes**

**Selection reason:** Naive Bayes assumes feature independence, leading to simple and efficient computation suitable for high-dimensional data. Although the independence assumption may not fully apply to CT radiomics features, Naive Bayes is valuable for preliminary analysis, particularly when quick results are needed.

**Advantages:**

- Simple and efficient: Fast computation, suitable for high-dimensional and small sample data, with minimal overfitting risk.
- Quick modeling: Suitable for preliminary analysis and rapid modeling.

**Disadvantages:**

- Independence assumption: The assumption of feature independence often does not hold in reality, affecting model performance.
- Sensitivity to Correlation: Performance significantly drops when the independence assumption is violated.
  1. **10. Partial Least Squares Regression for Generalized Linear Models (plsRglm)**

**Selection reason:** This approach employs partial least squares regression within the context of generalized linear models. It is particularly beneficial when dealing with many predictors exhibiting high multicollinearity. This technique combines elements of principal component analysis and canonical correlation analysis for model construction.

**Advantages:**

- Handles multicollinearity: Suitable for high-dimensional and small sample data.
- Improves predictive accuracy and interpretability: Performs well in improving prediction accuracy and interpretability.

**Disadvantages:**

- Limited non-linear capability: Less effective in handling non-linear relationships.
- Dependence on Sample Size: In cases with small sample sizes, plsRglm may be subject to significant random fluctuations, leading to unstable model results.
  1. **11. Linear Discriminant Analysis (LDA)**

**Selection reason:** LDA is a classification technique that establishes a linear decision boundary by fitting the data to class-conditional densities and using Bayes’ theorem for inference. It estimates the distributions of predictors independently within each category of the outcome variable and applies Bayes’ theorem to predict the probability of the outcome. LDA is employed in fields like statistics, pattern recognition, and machine learning to identify a linear combination of features that distinguishes or defines two or more classes.

**Advantages:**

- Simple and efficient: Easy to implement with high computational efficiency.
- Suitable for linearly separable data: Provides intuitive decision boundaries, aiding in understanding classification results.

**Disadvantages:**

- Limited non-linear capability: Less effective for non-linearly separable data.
- Sensitive to noise and outliers: More prone to noise and outliers.
  1. **12. Generalized Boosted Regression Modeling (GBM)**

**Selection reason:** GBM is a powerful ensemble learning algorithm that iteratively trains multiple weak classifiers to enhance overall model performance. It excels in capturing complex nonlinear relationships and interactions among various variables.

**Advantages:**

- Captures complex non-linear relationships: Improves robustness and accuracy by integrating multiple weak classifiers.
- Flexible parameter optimization: Allows parameter tuning to optimize model performance, providing great flexibility.

**Disadvantages:**

- Long training time: High computational cost.
- Overfitting risk: Prone to overfitting, necessitating careful parameter selection and model validation.
  1. **13. Logistic Regression**

**Selection reason:** Logistic regression is a Generalized Linear Model (GLM) primarily used for binary classification. By estimating the probability of an event, logistic regression can predict classification outcomes. Its core is the sigmoid function, which maps the linear combination of predictor variables to the [0, 1] interval, representing probability.

**Advantages:**

- Strong interpretability: The coefficients directly indicate each predictor variable's impact on the classification result, which is crucial for feature interpretation in medical research.
- Wide applicability: Extensively used for binary classification across various fields.
- High computational efficiency: Compared to other complex machine learning algorithms, logistic regression has low computational complexity and is suitable for large-scale datasets.
- Robustness and scalability: Handles different data scales well and effectively prevents overfitting with regularization techniques. It also demonstrates good robustness to missing data and outliers.

**Disadvantages:**

- Linear assumption: Assumes a linear relationship between predictor and response variables, which may not always hold, limiting model performance in cases with non-linear data.
- Feature independence assumption: Assumes predictors are independent, which may not be true, affecting the model's effectiveness.
- Sensitivity to outliers: Although somewhat robust, extreme outliers can significantly impact coefficient estimates.
- Limited multi-class handling: Primarily designed for binary classification, requiring extensions for multi-class problems, increasing model complexity.

**Supplementary Material S2**

AP+VP-Radscore = -0.091+ 1.317 * AP_original_glszm_HighGrayLevelZoneEmphasis

-3.488 * AP_log.sigma.5.0.mm.3D_firstorder_MeanAbsoluteDeviation

+0.783 * AP_wavelet.HHL_glszm_ZoneEntropy

+10.929 * AP_wavelet.LLL_gldm_SmallDependenceHighGrayLevelEmphasis

+0.354 * VP_log.sigma.4.0.mm.3D_glszm_SmallAreaHighGrayLevelEmphasis

-2.294 * VP_wavelet.LLH_glszm_SmallAreaHighGrayLevelEmphasis

+6.937 * VP_wavelet.LHL_glcm_ClusterShade

-1.827 * VP_wavelet.LHL_glcm_SumEntropy

+1.514 * VP_wavelet.HHL_glszm_ZoneEntropy

# Supplementary Figures and Tables

## Supplementary Tables

**Supplementary Table 1.** Algorithm combinations.

| **Algorithm combination** |
| --- |
| Enet [alpha=0.1] |
| Enet [alpha=0.2] |
| Enet [alpha=0.3] |
| Enet [alpha=0.4] |
| Enet [alpha=0.5] |
| Enet [alpha=0.6] |
| Enet [alpha=0.7] |
| Enet [alpha=0.8] |
| Enet [alpha=0.9] |
| GBM |
| glmBoost |
| glmBoost + Enet [alpha=0.1] |
| glmBoost + Enet [alpha=0.2] |
| glmBoost + Enet [alpha=0.3] |
| glmBoost + Enet [alpha=0.4] |
| glmBoost + Enet [alpha=0.5] |
| glmBoost + Enet [alpha=0.6] |
| glmBoost + Enet [alpha=0.7] |
| glmBoost + Enet [alpha=0.8] |
| glmBoost + Enet [alpha=0.9] |
| glmBoost + GBM |
| glmBoost + Lasso |
| glmBoost + LDA |
| glmBoost + plsRglm |
| glmBoost + Ridge |
| glmBoost + Stepglm [backward] |
| glmBoost + Stepglm [both] |
| glmBoost + Stepglm [forward] |
| glmBoost + SVM |
| glmBoost+NaiveBayes |
| glmBoost+XGBoost |
| Lasso + glmBoost |
| Lasso + LDA |
| Lasso + plsRglm |
| Lasso + Stepglm [backward] |
| Lasso + Stepglm [both] |
| Lasso + Stepglm [forward] |
| Lasso + SVM |
| Lasso+NaiveBayes |
| Lasso+XGBoost |
| Lasso+Logistic |
| LDA |
| NaiveBayes |
| plsRglm |
| RF |
| Ridge |
| Stepglm [backward] |
| Stepglm [backward] + Enet [alpha=0.1] |
| Stepglm [backward] + Enet [alpha=0.2] |
| Stepglm [backward] + Enet [alpha=0.3] |
| Stepglm [backward] + Enet [alpha=0.4] |
| Stepglm [backward] + Enet [alpha=0.5] |
| Stepglm [backward] + Enet [alpha=0.6] |
| Stepglm [backward] + Enet [alpha=0.7] |
| Stepglm [backward] + Enet [alpha=0.8] |
| Stepglm [backward] + Enet [alpha=0.9] |
| Stepglm [backward] + GBM |
| Stepglm [backward] + glmBoost |
| Stepglm [backward] + Lasso |
| Stepglm [backward] + plsRglm |
| Stepglm [backward] + Ridge |
| Stepglm [backward] + SVM |
| Stepglm [backward]+LDA |
| Stepglm [both] |
| Stepglm [both] + Enet [alpha=0.1] |
| Stepglm [both] + Enet [alpha=0.2] |
| Stepglm [both] + Enet [alpha=0.3] |
| Stepglm [both] + Enet [alpha=0.4] |
| Stepglm [both] + Enet [alpha=0.5] |
| Stepglm [both] + Enet [alpha=0.6] |
| Stepglm [both] + Enet [alpha=0.7] |
| Stepglm [both] + Enet [alpha=0.8] |
| Stepglm [both] + Enet [alpha=0.9] |
| Stepglm [both] + GBM |
| Stepglm [both] + glmBoost |
| Stepglm [both] + Lasso |
| Stepglm [both] + plsRglm |
| Stepglm [both] + Ridge |
| Stepglm [both] + SVM |
| Stepglm [both]+LDA |
| Stepglm [forward] |
| Stepglm[backward]+NaiveBayes |
| Stepglm[both]+NaiveBayes |
| SVM |
| XGBoost |

Note: Lasso, Least Absolute Shrinkage and Selection Operator; Enet, elastic net; Stepglm, Stepwise generalized linear model; SVM, support vector machine; glmBoost, boosted generalized linear model; LDA, Linear Discriminant Analysis; plsRglm, Partial Least Squares Regression for Generalized Linear Models; RF, random forest; GBM, generalised boosted regression modelling; XGBoost, Extreme Gradient Boosting; NaiveBayes, Naive Bayes.

**Supplementary Table 2.** Predictive performance for the proposed models in five-fold cross validation.

| Folds | Different models | Training cohort | | | |  |  |  |  |  |  | Validation cohort | | | |  |  |  |  |  |
| --- | --- | --- | --- | --- | --- | --- | --- | --- | --- | --- | --- | --- | --- | --- | --- | --- | --- | --- | --- | --- |
|  |  | AUC  (95%CI) | Sensitivity (%) | Specificity (%) | Accuracy (%) | F1 score  (%) | Recall  (%) | Precision  (%) | PPV  (%) | NPV  (%) |  | AUC  (95%CI) | Sensitivity (%) | Specificity (%) | Accuracy (%) | F1 score  (%) | Recall  (%) | Precision  (%) | PPV  (%) | NPV  (%) |
| Fold 1 | Clinical model | 0.685  (0.582-0.788) | 52.00 | 75.00 | 63.73 | 58.43 | 52.00 | 66.67 | 66.67 | 61.90 |  | 0.763  (0.577-0.949) | 69.23 | 61.54 | 65.38 | 66.67 | 69.23 | 64.29 | 64.29 | 66.67 |
|  | AP+VP-Radscore | 0.890  (0.829-0.951) | 80.00 | 76.92 | 78.43 | 78.43 | 80.00 | 76.92 | 76.92 | 80.00 |  | 0.941  (0.852-1.000) | 84.62 | 84.62 | 84.62 | 84.62 | 84.62 | 84.62 | 84.62 | 84.62 |
|  | Combined model | 0.904  (0.843-0.964) | 84.00 | 82.69 | 83.33 | 83.17 | 84.00 | 82.35 | 82.35 | 84.31 |  | 0.941  (0.858-1.000) | 84.62 | 69.23 | 76.92 | 78.57 | 84.62 | 73.33 | 73.33 | 81.82 |
| Fold 2 | Clinical model | 0.695  (0.594-0.796) | 58.00 | 71.15 | 64.71 | 61.70 | 58.00 | 65.91 | 65.91 | 63.79 |  | 0.716  (0.506-0.926) | 53.85 | 84.62 | 69.23 | 63.64 | 53.85 | 77.78 | 77.78 | 64.71 |
|  | AP+VP-Radscore | 0.916  (0.864-0.968) | 82.00 | 80.77 | 81.37 | 81.19 | 82.00 | 80.39 | 80.39 | 82.35 |  | 0.846  (0.693-1.000) | 69.23 | 84.62 | 76.92 | 75.00 | 69.23 | 81.82 | 81.82 | 73.33 |
|  | Combined model | 0.920  (0.870-0.971) | 86.00 | 76.92 | 81.37 | 81.90 | 86.00 | 78.18 | 78.18 | 85.11 |  | 0.864  (0.723-1.000) | 69.23 | 84.62 | 76.92 | 75.00 | 69.23 | 81.82 | 81.82 | 73.33 |
| Fold 3 | Clinical model | 0.715  (0.615-0.814) | 60.00 | 75.00 | 67.65 | 64.52 | 60.00 | 69.77 | 69.77 | 66.10 |  | 0.621  (0.396-0.846) | 46.15 | 69.23 | 57.69 | 52.17 | 46.15 | 60.00 | 60.00 | 56.25 |
|  | AP+VP-Radscore | 0.910  (0.855-0.965) | 84.00 | 78.85 | 81.37 | 81.55 | 84.00 | 79.25 | 79.25 | 83.67 |  | 0.893  (0.766-1.000) | 61.54 | 92.31 | 76.92 | 72.73 | 61.54 | 88.89 | 88.89 | 70.59 |
|  | Combined model | 0.913  (0.858-0.968) | 88.00 | 78.85 | 83.33 | 83.81 | 88.00 | 80.00 | 80.00 | 87.23 |  | 0.870  (0.731-1.000) | 69.23 | 84.62 | 76.92 | 75.00 | 69.23 | 81.82 | 81.82 | 73.33 |
| Fold 4 | Clinical model | 0.722  (0.625-0.820) | 60.78 | 73.08 | 66.99 | 64.58 | 60.78 | 68.89 | 68.89 | 65.52 |  | 0.609  (0.378-0.840) | 50.00 | 61.54 | 56.00 | 52.17 | 50.00 | 54.55 | 54.55 | 57.14 |
|  | AP+VP-Radscore | 0.905  (0.852-0.959) | 78.43 | 80.77 | 79.61 | 79.21 | 78.43 | 80.00 | 80.00 | 79.25 |  | 0.872  (0.718-1.000) | 100.00 | 61.54 | 80.00 | 82.76 | 100.00 | 70.59 | 70.59 | 100.00 |
|  | Combined model | 0.912  (0.860-0.964) | 80.39 | 78.85 | 79.61 | 79.61 | 80.39 | 78.85 | 78.85 | 80.39 |  | 0.904  (0.761-1.000) | 100.00 | 69.23 | 84.00 | 85.71 | 100.00 | 75.00 | 75.00 | 100.00 |
| Fold 5 | Clinical model | 0.691  (0.589-0.793) | 52.94 | 73.08 | 63.11 | 58.70 | 52.94 | 65.85 | 65.85 | 61.29 |  | 0.776  (0.586-0.965) | 50.00 | 84.62 | 68.00 | 60.00 | 50.00 | 75.00 | 75.00 | 64.71 |
|  | AP+VP-Radscore | 0.888  (0.827-0.949) | 76.47 | 78.85 | 77.67 | 77.23 | 76.47 | 78.00 | 78.00 | 77.36 |  | 0.968  (0.912-1.000) | 100.00 | 76.92 | 88.00 | 88.89 | 100.00 | 80.00 | 80.00 | 100.00 |
|  | Combined model | 0.897  (0.837-0.957) | 80.39 | 84.62 | 82.52 | 82.00 | 80.39 | 83.67 | 83.67 | 81.48 |  | 0.942  (0.847-1.000) | 100.00 | 76.92 | 88.00 | 88.89 | 100.00 | 80.00 | 80.00 | 100.00 |

## Supplementary Figures


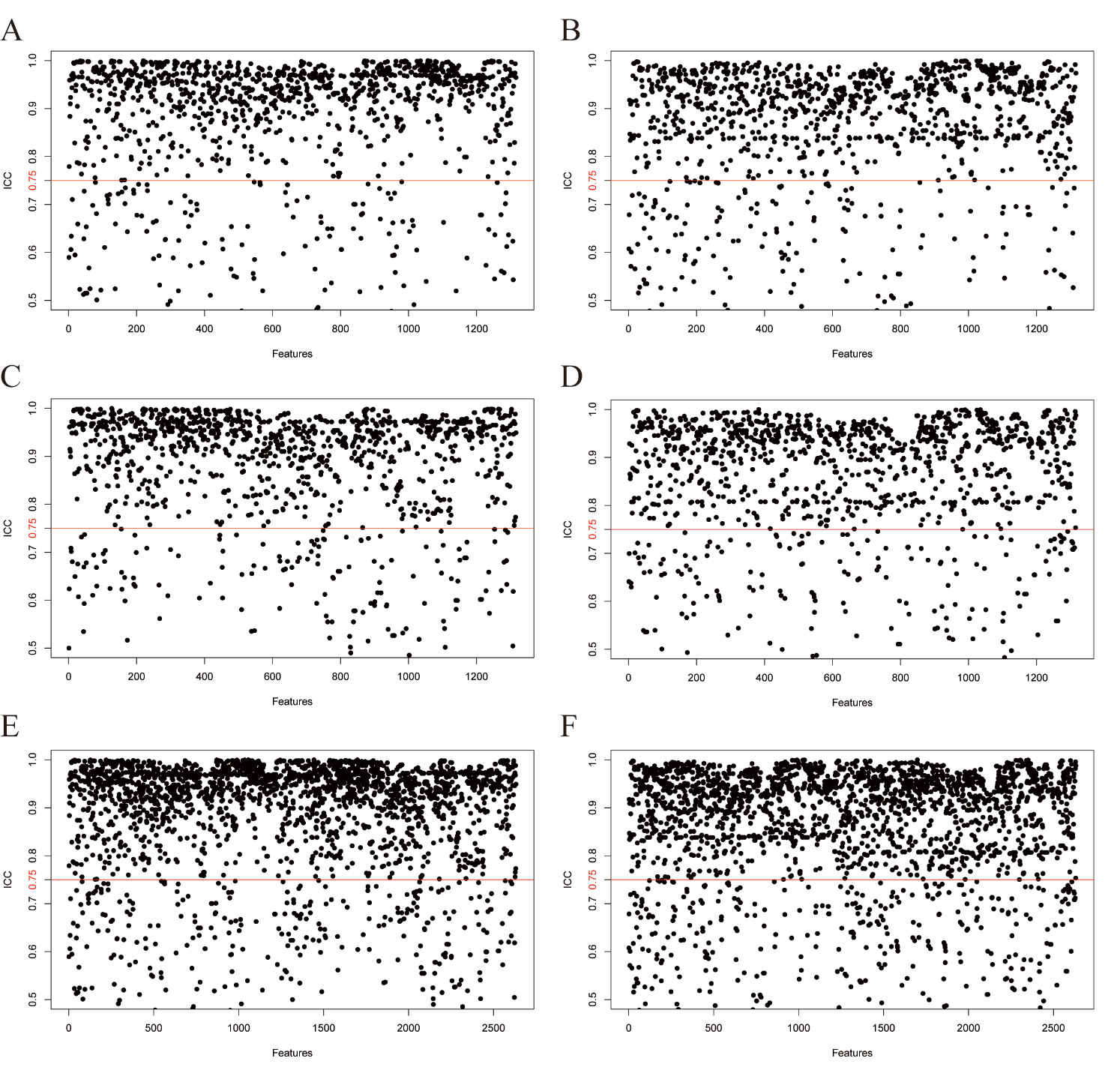


**Supplementary Figure 1.** Inter-observer and intra-observer agreements are based on the interclass correlation coefficient (ICC). (A) Intra-observer agreement in AP images. (B) Inter-observer agreement in AP images. (C) Intra-observer agreement in VP images. (D) Inter-observer agreement in VP images. (E) Intra-observer agreement in AP+VP images. (F) Inter-observer agreement in AP+VP images.


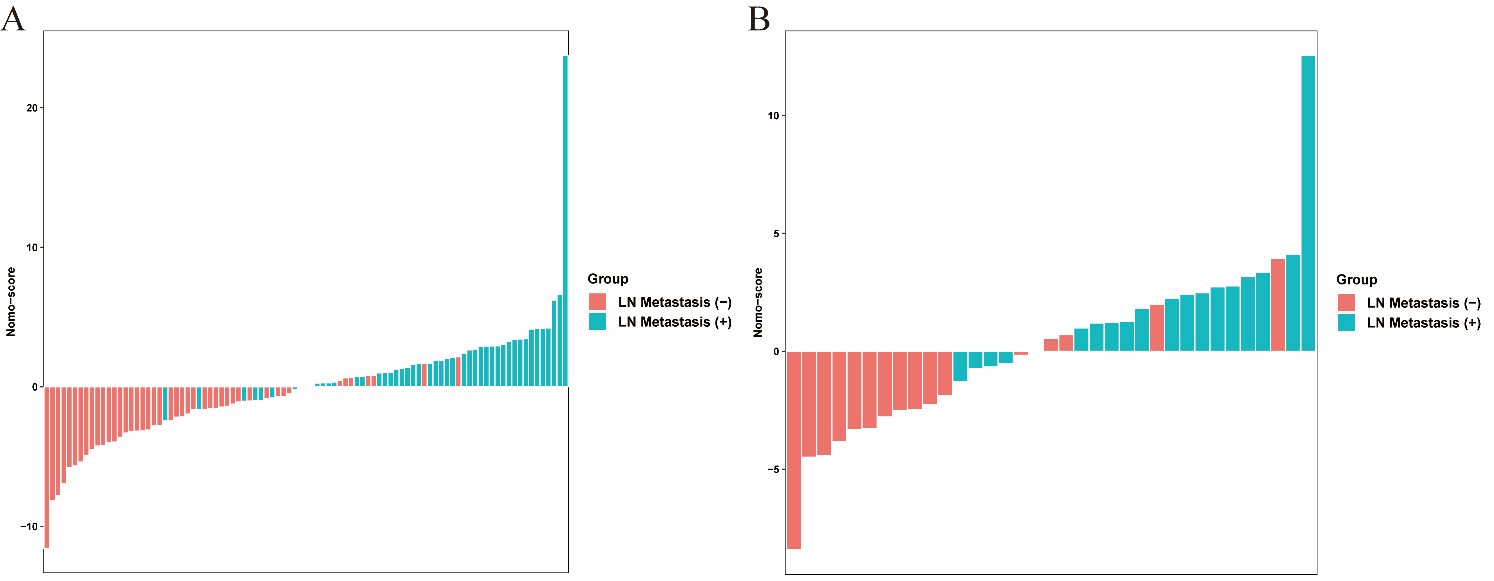


**Supplementary Figure 2.** The bar charts of Nomo-scores in the LN metastasis (-) and LN metastasis (+) groups. The bar charts displayed the Nomo-scores in the training (A) and validation (B) cohorts, respectively.


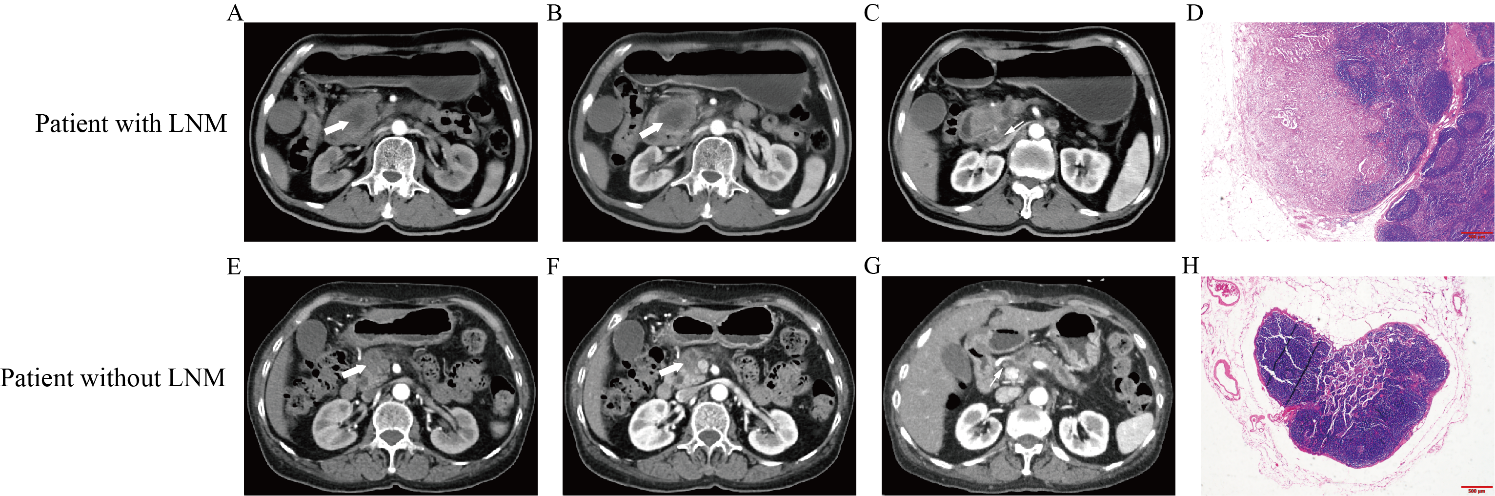


**Supplementary Figure 3.** Preoperative contrast-enhanced CT images and hematoxylin-eosin (H&E) staining pictures of two cases with PDAC. (A–D) A 73-year-old man with lymph node metastasis (magnification, ×4), the nomogram predicted that the probability of lymph node metastasis in this patient was 76.9%, indicating a high risk of lymph node metastasis. (E–H) A 73-year-old woman without lymph node metastasis (magnification, ×4), the nomogram predicted that the probability of lymph node metastasis in this patient was 18.9%, indicating a low risk of lymph node metastasis. The thick arrows are tumors, and the thin arrows are lymph nodes.
